# Modeling healthcare authorization and claim submissions using the openEHR dual-model approach

**DOI:** 10.1186/1472-6947-11-60

**Published:** 2011-10-12

**Authors:** Rigoleta DM Dias, Timothy W Cook, Sergio M Freire

**Affiliations:** 1 Centro de Análises de Sistemas Navais, Comando da Marinha, Ministério da Defesa, Programa de Pós-Graduação em Ciências Médicas, Universidade do Estado do Rio de Janeiro, Av. Prof Manuel de Abreu, 2° andar/LAMPADA, 20550-170, Rio de Janeiro, RJ, Brazil; 2 International Collaborator, Research Laboratory "Multilevel Healthcare Information Modelling", Universidade Federal Fluminense, Niterói, RJ, Brazil; 3 Programa de Pós-Graduação em Ciências Médicas, Universidade do Estado do Rio de Janeiro, Rio de Janeiro, RJ, Brazil

## Abstract

**Background:**

The TISS standard is a set of mandatory forms and electronic messages for healthcare authorization and claim submissions among healthcare plans and providers in Brazil. It is not based on formal models as the new generation of health informatics standards suggests. The objective of this paper is to model the TISS in terms of the openEHR archetype-based approach and integrate it into a patient-centered EHR architecture.

**Methods:**

Three approaches were adopted to model TISS. In the first approach, a set of archetypes was designed using ENTRY subclasses. In the second one, a set of archetypes was designed using exclusively ADMIN_ENTRY and CLUSTERs as their root classes. In the third approach, the openEHR ADMIN_ENTRY is extended with classes designed for authorization and claim submissions, and an ISM_TRANSITION attribute is added to the COMPOSITION class. Another set of archetypes was designed based on this model. For all three approaches, templates were designed to represent the TISS forms.

**Results:**

The archetypes based on the openEHR RM (Reference Model) can represent all TISS data structures. The extended model adds subclasses and an attribute to the COMPOSITION class to represent information on authorization and claim submissions. The archetypes based on all three approaches have similar structures, although rooted in different classes. The extended openEHR RM model is more semantically aligned with the concepts involved in a claim submission, but may disrupt interoperability with other systems and the current tools must be adapted to deal with it.

**Conclusions:**

Modeling the TISS standard by means of the openEHR approach makes it aligned with ISO recommendations and provides a solid foundation on which the TISS can evolve. Although there are few administrative archetypes available, the openEHR RM is expressive enough to represent the TISS standard. This paper focuses on the TISS but its results may be extended to other billing processes. A complete communication architecture to simulate the exchange of TISS data between systems according to the openEHR approach still needs to be designed and implemented.

## Background

Patient administrative data, such as insurance policies and billing processes, referrals, discharges and transfers, are needed to support safe, efficient, and effective healthcare delivery within both payer and provider organizations [1]. In Brazil, there is a significant difference between the healthcare billing process in the public system, called *Sistema Único de Saúde *(SUS) in Portuguese, and in the private one. In the SUS, the process is based on standardized information systems [2]. In the private sector it is relatively more complex due to the multiplicity of payers (around 1500 health plans) and providers (around 300, 000 including hospitals, laboratories, physicians and dentists) with different billing rules. For this reason, in 2005 the National Agency for Supplementary Health (ANS, in Portuguese) established a national standard for exchanging administrative healthcare data for the authorization and billing of both medical and dental services among health plans and providers, called TISS (*Troca de Informação em Saúde Suplementar*, in Portuguese) [3]. It is considered a major milestone in the discussion for unifying the billing process in the private sector.

Following the International Organization for Standardization/Technical Committee 215 (ISO/TC 215) for Health Informatics working group division [4], the TISS proposal comprises four types of standards: data structure, semantic content, communication and security. The data structure refers to the forms used in the authorization and billing process. The semantic content refers to the terminologies used to fill in the forms (still under development). The communication standard refers to the electronic messages for patient eligibility, authorization requests for consultations, tests, hospital or dental procedures, as well as for billing. The security issues are related to the privacy and confidentiality requirements for exchanging healthcare information.

A governance body coordinates the TISS evolution and through a review process the standard eventually undergoes some changes, such as including new attributes in the forms, changing their concepts or even creating new forms. For instance, new sets of clinical information to support authorizations and claims submissions are usually under discussion. Currently, the TISS does not support a multiple relationship between services and diagnoses. Changing this cardinality and adding new sets of clinical information definitely impacts on healthcare information systems that exchange TISS data. Although there is no underlying model for TISS data structures, the governance body intends to evolve the TISS to a patient-centered Electronic Health Record (EHR) policy and plans to establish an integrated claim and EHR model.

Standards for EHR are discussed in several organizations, such as ISO/TC215, Health Level 7 (HL7) [5], the European Committee for Standardization (CEN/TC 251) [6] and the openEHR Foundation [7]. In order to obtain fully functional and semantic interoperability among EHR information, the ISO 20514 technical report [8] establishes that it is necessary to standardize reference models, service interface models, domain-specific concept models (using archetypes and templates) and terminologies, paving the way for the adoption of a two-level modeling approach. Archetypes have been considered an appropriate solution for future-proof and interoperable medical data storage [9].

HL7 version 2 has a set of messaging specifications to support claims reimbursement using electronic exchange of health invoices [5]. The lack of a commonly used format for information exchange has led to the development of version 3, based on a formal information model called Reference Information Model (RIM) [10]. The RIM represents the core classes and attributes that will be required by the different messages in order to clarify the definitions and ensure that they are used consistently, but it does not represent a full EHR model [11-13].

The ISO 13606 series of standards [14-18], originally developed by CEN, defines a rigorous and stable information architecture for communicating part or all of the Electronic Health Record (EHR) of a single subject of care (a patient). It uses the dual model approach: a reference information model on the first level and an archetype model on the second one. However, the reference model is mainly for EHR communication and, like HL7, it is not a full EHR model [11-13].

The openEHR Foundation has defined a collaborative set of specifications for EHR systems also based on a two-level approach: information and knowledge [19]. The information level is represented by a generic reference model that includes both administrative and clinical entries. The knowledge level is represented by an archetype model where narrower concepts are specified. The openEHR specifications also include the service model that allows access to the data contained in the previous two models [20]. Its architecture can be adopted from a small information system to a full-fledged patient-centered shared solution [19,20].

This paper aims at re-designing the TISS standard according to the openEHR dual model approach in order to provide the TISS with a solid foundation on which to base its evolution. In this way, the set of archetypes can be used not only to communicate TISS messages but also can be used in openEHR-based EHR systems to store billing information and integrate them into an EHR architecture. The next section describes how the TISS standard and openEHR dual model approach have been used to propose a new methodology for the TISS. The results section presents an extension of the openEHR RM to deal with administrative authorization and claim submissions and the archetypes designed to represent the TISS standard. Then a discussion of these results, suggestions for future research and conclusions complete the paper.

## Methods

### The TISS structure

The TISS data structure refers to the paper forms used by providers to register healthcare events for the purpose of billing [21]. The forms contain demographic, administrative and clinical information. There are different forms used by medical providers to represent a consultation, a request for laboratory tests and hospitalization, laboratory tests and hospitalization summaries containing the services, the materials used and the staff involved. All forms can be sent electronically following a set of standardized electronic messages developed in Extended Markup Language (XML).

Every form contains a header with its identification and information about the healthcare provider, payer and patient. There are different types of administrative and clinical information. For instance, the form used for consultation contains information such as the diagnosis description and ICD-10 code, the indication of accident and the type of consultation. The one used for hospital summary contains information about the hospitalization being an elective or an emergency one, the ICD-10 code to classify diagnosis, procedures and tests, and medical staff involved. The one used for lab test request contains text fields for the test code and description and for diagnostic hypotheses. The forms used for dental treatment contain information about the procedures, the tooth identification, the face side and the type of care (treatment, radiology, orthodontics, and emergency).

The TISS billing process follows the traditional billing life cycle: request, authorization, claim, denied or completed. In the case of a refusal, it may be resubmitted and reanalyzed by the payer. However the values for each status are codified only in the message schemas and not in the forms. The same is the case for some attributes: billing date, insured identifiers (one for the healthcare provider and the other for the insurance company).

### The openEHR architecture

The openEHR Foundation's methodology is based on the two-level modeling approach: on the first level a common reference model (RM) is established, using a predefined set of classes that model the structure of the electronic record; and on the second level, specific concepts are established, by restricting the RM classes, in the form of archetypes, expressed in the Archetype Definition Language (ADL) [22], that can be translated to any language. For example, a restriction to the first level concept of "Observation" can be made by the blood pressure archetype which represents a description of all the information a clinician might want to report about it. Archetypes enable binding to different terminologies [23,24] and are designed to represent maximal data sets, that is, they are designed to represent concepts as generically as possible. Archetypes are then combined in templates in order to generate forms, messages, etc. The templates may further restrict the archetype elements and select the terminologies they will use in order to meet the requirements of the specific context they apply.

**Figure 1:**
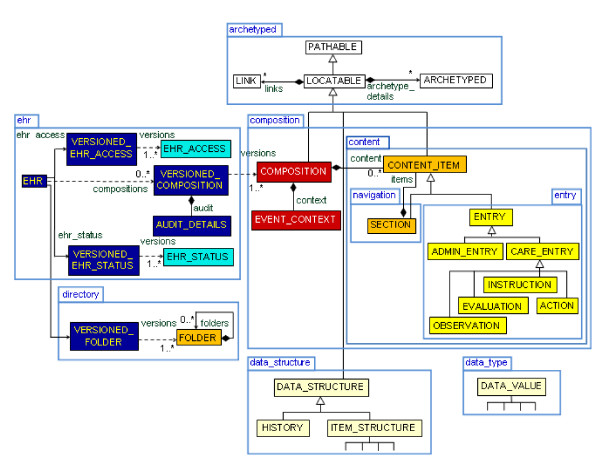
**openEHR reference model**.

Figure 1 shows a simplified view of the openEHR reference model. In summary, an EHR is a set of compositions (COMPOSITION) whose contents (ENTRY) may be of a clinical or an administrative type. The entries can be organized in sections (SECTION). The ENTRY class is divided into two categories: healthcare data (CARE_ENTRY) and administrative data (ADMIN_ENTRY). The CARE_ENTRY class covers the entire process of patient care and is divided into OBSERVATION, EVALUATION, INSTRUCTION and ACTION that actually relates to past, present and future events. The ADMIN_ENTRY class refers to the administrative data filled in by doctors or nurses. For instance, it can represent admission, scheduling, and requests. Differently from the CARE_ENTRY class, it is a generic class without any other conceptual subclasses. Every data value is recorded in an ELEMENT object. ELEMENTs may be organized in structures such as tables (ITEM_TABLE), trees (ITEM_TREE), lists (ITEM_LIST) and a single ELEMENT (ITEM_SINGLE).

The openEHR platform, including the RM, archetypes and templates, represents healthcare information with full meaning and interoperability [25-31]. In order to help users design good quality archetypes in a friendly and graphical way, there are some free tools available [32-34]. The archetypes already designed and being internationally revised are available in a repository called the Clinical Knowledge Manager (CKM) [35].

### Silverston's models for healthcare claim submission

Several authors have proposed analytic models for various areas such as finance, telecommunications, commerce, tourism, insurance, healthcare, etc [36-39]. These models are a result of abstracting patterns from experience with a number of projects and their aim is to reduce development time and software costs, and provide high quality system designs. Developers can adapt the models to their needs and establish business rules.

**Figure 2:**
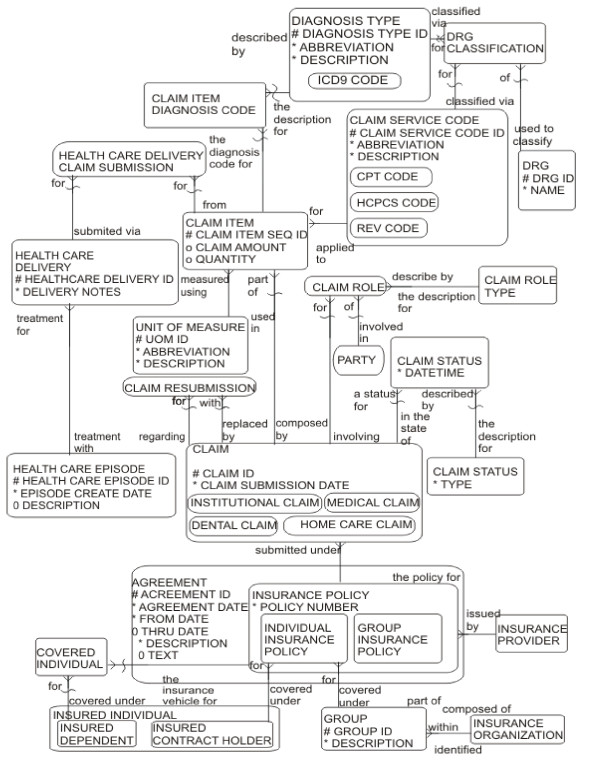
**Silverton's relational model for healthcare claims**. Reprinted with permission of John Wiley & Sons, Inc.

Silverston's relational model for healthcare claims submission [36] is applicable in this context and is presented in Figure 2. Only the part of the model related to the billing process will be explained here. Tables that deal with agreement settlement between a healthcare provider and a healthcare plan are outside the scope of this paper. Claims (CLAIM) are sub-typed into institutional, dental, medical and home care. Each claim has a status (CLAIM STATUS) and consists of one or more items (CLAIM ITEM). Each item is related to a service provided (CLAIM SERVICE CODE), one or more diagnoses (CLAIM ITEM DIAGNOSIS CODE) and to a health care delivery (HEALTH CARE DELIVERY) through HEALTH CARE DELIVERY CLAIM SUBMISSION. A health care delivery is any services (consultations, tests, procedures, drug administration and so on) provided during a health care visit for one health care episode (HEALTH CARE EPISODE). For example, a physical therapy as a service may be related to two diagnoses of a fractured arm and a fractured wrist. Observe that a service code may be expressed in a different terminology of that used to express the corresponding health care delivery. A claim may have several statuses, for example: "submitted"; "pending"; "denied"; "returned for correction" and "completed".

The demographic entities that take part in a billing process are represented in the PARTY, CLAIM_ROLE and CLAIM_TYPE tables.

The openEHR clinical RM has classes that represent concepts similar to the ones in Silverston's model: EVALUATION - DIAGNOSIS; ACTION - SERVICE_CODE/HEALTH_CARE_DELIVERY; COMPOSITION - CLAIM. Relationships between the above mentioned classes in openEHR may be expressed by the class LINK. For the demographic entities, the openEHR demographic RM has the classes PARTY and ROLE, related to the PARTY and CLAIM_ROLE tables, respectively. Details of each specific claim are expressed in archetypes and templates.

A set of archetypes was designed, using the Ocean Archetype Editor [32], to represent each section of the TISS forms: rooted in class EVALUATION containing elements related to the patient's clinical condition and services requested, rooted in ACTION for services performed, and rooted in ADMIN_ENTRY for administrative information. Then a set of templates were designed with Ocean Template Designer [40] to represent each TISS claim by combining the above set of archetypes.

As an alternative, another set of archetypes was designed with similar information to the archetypes based on EVALUATION and ACTION above, but rooted in CLUSTER, and an ADMIN_ENTRY rooted archetype which, besides administrative information, has several slots for the cluster-based archetypes. Again, a set of templates was built based on this second set of archetypes.

A third possibility, partly suggested by Silverston's model, is to extend openEHR RM ADMIN_ENTRY classes with subtypes that represent the several TISS claims, add some fixed attributes that applies to all TISS submissions, and add an attribute to represent the submission status.

A new set of archetypes was designed based on this model, using a text editor, since the tools available do not support this extended RM.

### Demographic Information

In order to represent the demographic data of patients, healthcare plans and healthcare providers, the demographic archetypes available in the openEHR repository [35] may be used since they provide all data structures that the TISS needs.

The openEHR RM has several classes with attributes of the PARTY_PROXY type that refer to demographic entities. Since the billing process involves the exchange of information between different systems, when referring to demographic entities, the PARTY_IDENTIFIED of PARTY_PROXY subclass needs to be used, as it has the identifier attribute which can be used to store the identifier of the entity in the target system. For instance, these identifiers may refer to the Brazilian Unique National Identifier for Health Care Providers, the health care plan number or the patient number in the health care plan. The attributes subject, provider and other_participants in the ENTRY class may be used to refer to entities involved in the billing process, namely the patient, the clinician or administrative staff who provided the information, and other participants (the insurance company, the person responsible for managing the billing process in the healthcare provider, etc).

### Simulation

As a proof of the concept, the openEHR RM java implementation was leveraged [41] in order to allow the implementation of the new classes in the extended RM, the extract package was implemented in order to allow the communication of EHR extracts, and a simple REST based web-service was also implemented in order to simulate the exchange of TISS extracts between a healthcare provider and an insurance company.

## Results

### Archetypes based on ENTRY(ies) subtypes

Table 1 shows ten archetypes designed to represent the sections that compose each block of TISS's forms, rooted according to the kind of information in the respective section. A composition archetype has a slot that accepts all the archetypes mentioned in Table 1. Templates were built to compose each of TISS forms using the composition archetype, selecting the respective archetypes, and excluding those elements that are not part of the form. Moreover, local codes were added in the templates to bind the corresponding values to local terminologies. In total, eleven templates were designed corresponding to the following forms: individual claim, consultation claim, admission request, admission authorization, admission claim, tests and procedures request, tests and procedures authorization, tests and procedures claim, dental evaluation, dental claim and other charges.

**Table 1:** Archetypes for the TISS standard

Archetypes	Information contained in the archetype
openEHR-EHR-EVALUATION. patient_evaluation.v1	**ELEMENTs**: clinical indication, type of disease, duration of disease, accident indication, main diagnosis, secondary diagnoses, cause of death, death certificate number. **CLUSTER (obstetrics**): gestation, abortion, pregnancy-related problems, puerperium complications, neonatal assistance, neonatal complication, low birthweight, cesarean section, normal delivery, maternal death, number of living births (living term births, premature living births, still births, early neonatal deaths, late neonatal deaths). **CLUSTER (planned services)**
	**ELEMENTs**: type of procedure, procedure, quantity requested, quantity authorized **CLUSTER **(**orthoses and prostheses): **vendor, unit cost.

openEHR-EHR-EVALUATION. odontologic_evaluation.v1	**ELEMENTs**: periodontal disease, alterations in soft tissues. **CLUSTER **(initial status): tooth, status

openEHR-EHR-ACTION.claim_services.v1	**ELEMENTs**: type of procedure, procedure, quantity authorized, quantity performed, access, technique, vendor, unit cost, total cost, percent of reduction/addition, deduction, tooth, face.

openEHR-CLUSTER.admission.v1	**ELEMENTs: **type of admission, probable date of admission, hospital service, admission regime, requested number of days, authorized number of days, type of accommodation, date of admission, discharge date.

openEHR-CLUSTER.total_costs.v1	**ELEMENTs: **total costs, total rent, total drugs, total materials, total hospital stay, total gases, total other rates, total procedures, total medicinal gases, Total deduction.

openEHR-EHR- ADMIN_ENTRY.professional_claim.v1	**ELEMENTs: **original submission, submission ID, submission date, validity, billing date, type of claim, type of encounter, date of encounter, discharge reason, further action, status, comments **SLOT for CLUSTER total_costs**.

openEHR-EHR- ADMIN_ENTRY.institutional_claim.v1	**ELEMENTs**: original submission, submission ID, submission date, billing date, type of claim, type of encounter, date of encounter, type of claim, discharge reason, further action, status, comments. **SLOT for CLUSTERs admission and total_costs**

openEHR-EHR- ADMIN_ENTRY. authorization.v1	**ELEMENTs**: original submission, submission ID, submission date, validity, authorization date, authorization number, type of encounter, date of encounter, status, comments. **SLOT for CLUSTER admission**

openEHR-EHR-ADMIN_ENTRY.authorization_request.v1	**ELEMENTs**: submission ID, submission date, status, type of encounter, date of encounter, comments. **SLOT for CLUSTER admission**

openEHR-EHR-COMPOSITION.tiss_claim.v1	**Archetype slots **that include each of the archetypes above.

These archetypes are based on the openEHR RM.

For illustration purposes the definition section of the archetype for the institutional claim concept based on the ADMIN_ENTRY class (openEHR-EHR-ADMIN_ENTRY.institutional_claim.v1) is shown below:

ADMIN_ENTRY[at0000] matches { -- claim header

data matches {

ITEM_TREE[at0001] matches { -- components

items cardinality matches {0..*; unordered} matches {

ELEMENT[at0002] occurrences matches {0..*} matches { -- submission ID

value matches { DV_TEXT matches {*} } }

ELEMENT[at0003] occurrences matches {0..*} matches { -- original submission

value matches { DV_TEXT matches {*} } }

ELEMENT[at0004] occurrences matches {0..1} matches { -- submission Date

value matches { DV_TEXT matches {*} } }

ELEMENT[at0005] occurrences matches {0..1} matches { -- type of encounter

value matches { DV_TEXT matches {*} } }

ELEMENT[at0006] occurrences matches {0..1} matches { -- date of encounter

value matches {DV_DATE_TIME matches {*} } }

ELEMENT[at0007] occurrences matches {0..1} matches { -- type of claim

value matches { DV_TEXT matches {*} } }

ELEMENT[at0008] occurrences matches {0..1} matches { -- billing date

value matches { DV_TEXT matches {*} } }

ELEMENT[at0009] occurrences matches {0..1} matches { -- status

value matches { DV_TEXT matches {*} } }

ELEMENT[at0010] occurrences matches {0..1} matches { -- discharge reason

value matches { DV_TEXT matches {*} } }

ELEMENT[at0011] occurrences matches {0..1} matches { -- further action

value matches { DV_TEXT matches {*} } }

ELEMENT[at0012] occurrences matches {0..1} matches { -- comments

value matches { DV_TEXT matches {*} } }

allow_archetype CLUSTER[at0015] occurrences matches {0..*} matches {

include archetype_id/value matches {

openEHR-EHR-CLUSTER.admission.v1 |

openEHR-EHR-CLUSTER.costs.v1 } }

### Archetypes based on ADMIN_ENTRY and CLUSTERS

The archetypes based on ADMIN_ENTRY and CLUSTERs have similar contents to those presented in Table 1, with the following differences: they are rooted in the CLUSTER class while the previous ones were rooted in the CARE_ENTRY subclasses; the archetypes rooted in ADMIN_ENTRY have a slot that accepts the other CLUSTER archetypes, besides the content of the original ADMIN_ENTRY archetype; some elements are included in the archetypes to allow attributes that are presented in the CARE_ENTRY subclasses, and therefore were not included in the previous archetypes; and finally the composition archetype has a slot that accepts the ADMIN_ENTRY rooted archetype. The templates were similarly designed, this time including the ADMIN_ENTRY archetype and the relevant CLUSTER archetypes.

### openEHR extended RM

**Figure 3:**
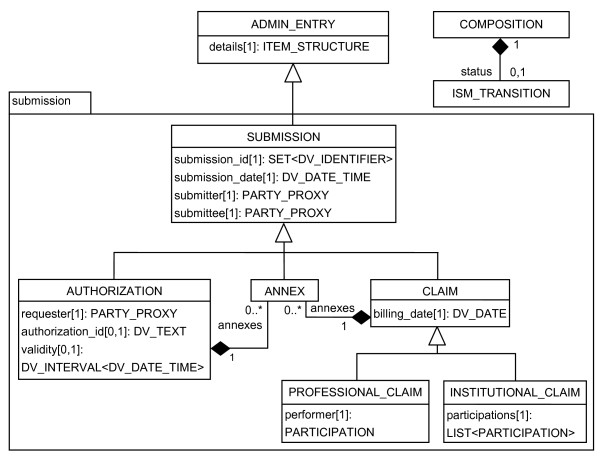
**The openEHR extended RM for authorization and claim submissions**.

Figure 3 shows the openEHR extended RM. A class named SUBMISSION is defined to represent concepts related to the process of sending and receiving authorization, claims or an annex. The SUBMISSION class contains fixed attributes that are common to all types of authorization and claims, such as submission_id, submission_date, submitter (provider) and submittee (payer).

A claim is a submission that may be either a professional or an institutional one (hospital, laboratory or clinic) and has a billing_date attribute. A health professional claim has a performer, which can be a physician, dentist, physiotherapist etc. An institutional claim contains a list of professionals. An authorization may be required to carry out a medical or dental procedure. Any healthcare professional can be represented by the requester attribute. A response to an authorization request is given an authorization_id and a period of validity. An annex is a complement to a submission when necessary. For example, an annex can be a request for a clinical report made by the payer to authorize chemotherapy.

### Archetypes based on the extended ADMIN_ENTRY class

Table 2 shows a set of archetypes, designed to model TISS forms according to the extended openEHR RM. They have similar contents to those presented in Table 1, with the following differences: the administrative archetypes are rooted in AUTHORIZATION, PROFESSIONAL_CLAIM, and INSTITUTIONAL_CLAIM classes; and several elements, such as date of bill, submission ID, submission date and original submission, were removed from the corresponding ADMIN_ENTRY archetype because they were included in the reference model. The templates follow the same principles as shown above. All sets of archetypes may be obtained from the authors of the article.

**Table 2:** Archetypes for the TISS standard

Archetypes	Information contained in the archetype
openEHR-EHR-EVALUATION.patient_evaluation.v1	Similar to corresponding archetype in table 1

openEHR-EHR-EVALUATION.odontologic_evaluation.v1	Similar to corresponding archetype in table 1

openEHR-EHR-ACTION.claim_services.v1	Similar to corresponding archetype in table 1

openEHR-CLUSTER.admission.v1	Similar to corresponding archetype in table 1

openEHR-CLUSTER.total_costs.v1	Similar to corresponding archetype in table 1

openEHR-EHR-PROFESSIONAL_CLAIM.professional_claim.v1	**ELEMENTs: **type of encounter, date of encounter, discharge reason, further action, comments **SLOT for CLUSTER total_costs**.

openEHR-EHR-INSTITUTIONAL_CLAIM.institutional_claim.v1	**ELEMENTs**: type of encounter, date of encounter, type of claim, discharge reason, further action, comments. **SLOT for CLUSTERs admission and total_costs**

openEHR-EHR-AUTHORIZATION.authorization.v1	**ELEMENTs**: type of encounter, date of encounter, comments. **SLOT for CLUSTER admission**

openEHR-EHR-AUTHORIZATION.authorization_request.v1	**ELEMENTs**: type of encounter, date of encounter, comments. **SLOT for CLUSTER admission**

openEHR-EHR-COMPOSITION.tiss_claim.v1	**Archetype slots **that include each of the archetypes above.

These archetypes are based on the extended openEHR RM.

For comparison purposes, below is the definition section of the archetype for the institutional claim concept, now based on the INSTITUTIONAL_CLAIM class (openEHR-EHR-INSTITUTIONAL_CLAIM.institutional_claim.v1). Comparing with the same concept expressed with the openEHR RM, it requires less elements which are now part of the extended RM:

INSTITUTIONAL_CLAIM[at0000] matches { -- claim header

data matches {

ITEM_TREE[at0001] matches { -- components

items cardinality matches {0..*; unordered} matches {

ELEMENT[at0002] occurrences matches {0..1} matches { -- type of encounter

value matches { DV_TEXT matches {*} } }

ELEMENT[at0003] occurrences matches {0..1} matches { -- date of encounter

value matches {DV_DATE_TIME matches {*} } }

ELEMENT[at0004] occurrences matches {0..1} matches { -- type of claim

value matches { DV_TEXT matches {*} } }

ELEMENT[at0005] occurrences matches {0..1} matches { -- status

value matches { DV_TEXT matches {*} } }

ELEMENT[at0006] occurrences matches {0..1} matches { -- discharge reason

value matches { DV_TEXT matches {*} } }

ELEMENT[at0007] occurrences matches {0..1} matches { -- further action

value matches { DV_TEXT matches {*} } }

ELEMENT[at0008] occurrences matches {0..1} matches { -- comments

value matches { DV_TEXT matches {*} } }

allow_archetype CLUSTER[at0010] occurrences matches {0..*} matches {

include archetype_id/value matches {

openEHR-EHR-CLUSTER.admission.v1 |

openEHR-EHR-CLUSTER.costs.v1 } }

### Model Implementation

The classes of the extended model were implemented based on the Java openEHR RM implementation, SUBMISSION being a specialization of ADMIN_ENTRY. In order to simulate authorization requests and authorization responses, a couple of composition objects were created according to the structure of the admission archetype, grouped in an EXTRACT, and sent to another target system, which upon receipt of the extract, processed the responses, wrapped them in another EXTRACT, and sent them back to the source system. The communication is based on a simple REST based web service, where each resource was identified by the/organizationID/{resource_id} path, using GET, POST, PUT, and DELETE as defined in the REST architecture. The web services were created using the Netbeans 7.0 IDE [42]. However, in a real scenario, data would be obtained through a graphical user interface and communicated to the target system by means of a set of standardized web-services. This architecture communication remains to be established.

## Discussion

### Modeling the TISS using the openEHR approach

Although openEHR has as its main focus the modeling of the clinical information for the EHR, its ADMIN-ENTRY class, together with all other classes in the RM, provides a basis to design archetypes that meet the requirements of the TISS data structures. Designing the TISS according to the openEHR dual-model approach aligns it with the interoperability recommendation of ISO technical report [8]. By using a stable RM and concepts expressed through archetypes, every change to the standard is carried out by simply designing new archetypes, or by specializing or creating new versions of the existing ones. Systems that adopt the openEHR architecture are supposed to accommodate the standard evolution much more easily than those that follow the traditional one-level model [22,43-47].

### Extended openEHR RM

In the extended RM classes some attributes refer to demographic entities by means of the classes: PARTY_PROXY: submitter; submittee in SUBMISSION; performer in PROFESSIONAL_CLAIM; participants in INSTITUTIONAL_CLAIM; and requester in AUTHORIZATION. In fact, they would not be necessary, since the attribute other_participations in class ENTRY could be used instead. On the other hand this attribute illustrates the clinical-oriented aspect of the openEHR RM model. In the case of adhering to the extended RM model, the attribute other_participations could be moved down the hierarchy to the CARE_ENTRY class so that the admin models would be free to specify the attributes that semantically better describe their demographic entities.

### openEHR RM versus extended openEHR RM

Table 3 presents a summary of the issues that may arise when comparing the basic openEHR RM with the extended RM proposed in this article.

**Table 3:** Comparison of the reference models for developing TISS archetypes

Criteria	Original openEHR RM	Extended openEHR RM
Tooling support	The present tools support it	Support for it must be implemented

Interoperability	Compatibility with openEHR-based systems	Non-compatibility with current openEHR-based system

Semantic Interpretation	Although biased to the clinical content, it may be used to represent administrative content related to authorization and claim submissions	Add subclasses and attributes aligned with the process of authorization and claim submissions

There are some open source tools that support the design of archetypes based on openEHR RM [32-34]. Basing the TISS archetypes on the current openEHR RM model does not require any changes in the current tools and it maintains interoperability with openEHR-based systems.

Using the extended RM will require the evolution of current tools and may disrupt interoperability with the current systems. This argument calls for adhering to the openEHR RM as long as there are no stronger reasons to move to another alternative. The openEHR Foundation focus is mainly in the development of specifications for building what are called future-proof EHR systems. At present there are still few system implementations based on openEHR, mainly dealing with the clinical model [46-49]. This reflects in slower developments in the demographic and administrative areas and also in the communication of EHR extracts. The specification for Extracts communication, for example, is still under development and there have been changes in its model with reflection in other packages [50]. This is illustrated by the introduction of two subclasses of the PARTICIPATION class. As more experience is gained with this approach and with the demographic and admin models, new requirements may emerge that might challenge the current RM. As a consequence, changes in the openEHR ecosystem may occur in the future. For this reason the tools will have to be designed considering the eventual evolution of the RM.

As illustrated by the definition sections of the archetypes for the institutional claim concept, and the inspection of tables 1 and 2, there are very little differences between the archetypes based on the openEHR or on the extended openEHR RM. The administrative archetypes based on the extended RM do not need to specify several elements that are now part of the RM. Other allowed values for the attributes of ISM_TRANSITION have to be defined to represent all the submission statuses, an attribute of COMPOSITION in the extended RM.

The proposed enhanced RM defines administrative concepts such as "authorization", "professional-claim" or "institutional-claim" in a similar manner to the CARE_ENTRY subclasses that deal with clinical generic concepts such as observation, evaluation, instruction and action. From those concepts, different types of claims can be structured. It is possible that in the future a similar rationale may arise when using openEHR related to other administrative aspects, such as scheduling and admission process, for instance.

The extended RM adds more semantics to the administrative content of the EHR, which arguably leads to an easier interpretation by modelers and developers.

In summary, a pragmatic perspective and the requirement for interoperability tend to favor the adherence to the current openEHR model. On the other hand, an aesthetic point of view favors the extended RM.

### Archetypes review and validation

The archetypes designed to represent the TISS based on the current openEHR RM or on the extended openEHR RM need to be internationally revised. The openEHR RM may possibly be used with other billing systems since it provides a common model that supports the restriction by archetypes to suit the needs of such systems. In Brazil, for example, an inspection of the forms used in the public healthcare billing systems revealed that their archetypes can be easily designed. Contrary to the clinical archetypes and even the demographic ones, the archetypes proposed here will probably not be reused in other countries due to the special characteristics of their billing processes. However, as more archetypes for billing processes are designed, it is possible that commonalities between them may be found and a set of common archetypes be built, with specializations taking care of the local needs. There should be rules for defining, managing and disseminating the archetypes in a public repository, like CKM, so that both sending and receiving information systems can access them [28,51,52].

### Future work

The authors envision two ways to continue this research. One is to design and implement a complete communication architecture to simulate the exchange of TISS data between systems according to the openEHR approach. The other one is to design archetypes to represent Brazilian public healthcare billing data based on the enhanced openEHR RM, and propose a unifying healthcare billing concepts in Brazil in order to make the current billing systems fully semantically interoperable.

## Conclusions

Modeling the TISS standard by means of the openEHR archetype-based approach aligns it with ISO recommendations and provides a solid foundation on which the TISS may base its evolution. Although the openEHR RM has a small emphasis on administrative tasks with few administrative archetypes available, it is expressive enough to represent the TISS standard. The extended openEHR RM model is more semantically aligned with the concepts involved in authorization and claim submissions. Although this paper focuses on the TISS standard, its results can be extended to other billing processes. A complete communication architecture to simulate the exchange of TISS data between systems according to the openEHR approach still needs to be designed and implemented.

## Competing interests

The authors declare that they have no competing interests.

## Authors' contributions

RDMD and SMF contributed to the modeling, archetypes design and the writing of the manuscript. TWC contributed to the modeling and the writing of the manuscript. All authors have read and approved the final version of the manuscript.

## Pre-publication history

The pre-publication history for this paper can be accessed here:

http://www.biomedcentral.com/1472-6947/11/60/prepub
